# Epidemiology, clinical outcomes and risk factors for mortality and severe postoperative complications in cardiac surgery patients admitted to intensive care units in Brazil: a multicentre observational study

**DOI:** 10.1016/j.bjao.2026.100580

**Published:** 2026-08-12

**Authors:** BraSIS 2 Study Group, Renato Carneiro de Freitas Chaves, Nair Naiara Barros de Vasconcelos, Carolina de Moraes Pellegrino, Anna Clara Legal, Guilherme Martins de Souza, Veronica Neves Fialho Queiroz, Carmen Silvia Valente Barbas, Flávio Takaoka, Ricardo Luiz Cordioli, Sandrigo Mangini, Fabio de Vasconcelos Papa, Hélio Penna Guimarães, Ary Serpa Neto, Tiago Mendonça dos Santos, Rogerio da Hora Passos, Frederico Toledo Campo Dall’Orto, Eric Benedet Lineburger, Vinícius Caldeira Quintão, Lucas Tramujas, Caio Vinicius Gouvea Jaoude, Karlos Alexandre de Sousa Vilarinho, Brenno Cardoso Gomes, Viviane Cordeiro Veiga, Erick César de Farias Albuquerque, Ricardo Reinaldo Bergo, Mauricio de Castro Gomes, Gislayne Rogante Ribeiro, Luis Guilherme Patricio, Mariuska Perez Apolonio, Júlia Turra Reichert, Felipe Fernandes Ronsoni, Ysys Bittencourt Martins Camargo, Guilherme Hentges Fusiger, Tales Ivan Freitas dos Santos, Igor Almeida Cunha, Kellen Nazário Pereira, Andre Gulinelli, Eduardo Paolinelli, Fabio Barlem Hohmann, Felipe Galdino, Felipe Vianna, Luciano Ribeiro Pereira Silva, Roberto Rabello Filho, Thais Dias Midega, Vinicius Barbosa Galindo, Maria José Carvalho Carmona, Alexandra Patrícia Zilli Vieira Pharm, Maxim Goncharov, Matheus Ligouri, Marcelo Luz Pereira Romano, Guilherme Nebó e Jambor, Guilherme Benevenuto Hasebe, Lara Beatriz Alves de Melo, Orlando Petrucci Junior, Matheus da Silva Passos, Gustavo Lenci Marques, Caroline Dourado Gomes, Tom Pollard, Adriano José Pereira, Thiago Domingos Corrêa, MD.PhD. João Manoel Silva Júnior

**Affiliations:** 1 Einstein Hospital Israelita, São Paulo, SP, Brazil; 2 Takaoka institute, São Paulo, SP, Brazil; 3 University of Toronto, Toronto, Canada; 4 University of Melbourne, Austin Hospital, Melbourne, Australia; 5 Hospital Maternidade e Pronto Socorro Santa Lúcia, Minas Gerais, MG, Brazil; 6 Hospital São José, Criciúma, SC, Brazil; 7 Academic Research Organization, Instituto do Coração, Hospital das Clínicas HCFMUSP, Faculdade de Medicina, Universidade de São Paulo, SP, Brazil; 8 Instituto de Pesquisa do Hcor, São Paulo, SP, Brazil; 9 Instituto de Assistência Médica ao Servidor Público Estadual, São Paulo, SP, Brazil; 10 Universidade Estadual de Campinas, Campinas, SP, Brazil; 11 Hospital de Clínicas da Universidade Federal do Paraná, Paraná, PR, Brazil; 12 BP - A Beneficência Portuguesa de São Paulo, São Paulo, SP, Brazil; 13 Hospital Alberto Urquiza Wanderley, João Pessoa, PB, Brazil; 14 Massachusetts Institute of Technology, Cambridge, MA, United States

**Keywords:** anaesthesia, cardiovascular surgery, critical care, epidemiology, mortality

## Abstract

**Background:**

Cardiac surgery carries substantial risks of perioperative complications and mortality; however, outcome data from low- and middle-income countries are scarce. This prospective observational cohort study conducted across 11 Brazilian hospitals aimed to determine the incidence of early mortality and major postoperative complications in patients undergoing cardiac surgery in Brazil.

**Methods:**

Adults (≥18 yr) undergoing coronary, valvular, or percutaneous cardiac surgery requiring postoperative ICU admission, excluding patients under exclusive palliative care, device implantation only, or moribund before surgery. Risk factors and incidence of mortality or severe postoperative complications within the first 3 postoperative days or until ICU discharge (whichever occurs first). Risk factors were assessed using generalised linear mixed-effects models, reported as odds ratios with 95% confidence intervals.

**Results:**

Between September 2023 and March 2025, 528 patients were enrolled. Among 528 patients (91% open-heart, mainly coronary artery bypass grafting and valve replacements), 170 (32%) experienced early mortality or severe postoperative complications, most within the first 24 h. The most frequent complications were severe haemodynamic instability (126, 24%), and acute respiratory distress syndrome (71, 13%). Most events occurred on the day of ICU admission (132 of 170, 78%). Risk factors of the primary outcome were prior stroke (3.22 [1.02–10.17], *P*=0.046), preoperative thrombocytopenia (2.83 [1.46–5.48], *P*=0.002), open-heart surgery (6.34 [1.66–24.21], *P*=0.007), biological valves (3.94 [1.18–11.31] *P*=0.026). Early postoperative markers of clinical deterioration were higher Sequential Organ Failure Assessment score on day 0 (1.35 [1.19–1.53], *P*<0.001), lower arterial pH on day 0 (0.53 [0.34–0.83], *P*=0.006), and vasoplegia (15.35 [6.05–38.94], *P*<0.001).

**Conclusions:**

In this multicentre cohort of cardiac surgery patients in Brazil, one in three experienced early mortality or severe postoperative complications, mostly within the first 24 h. Identified predictors of early postoperative deterioration may help guide perioperative strategies and underscore the need for developing prognostic models adapted to local contexts.

**Clinical trial registration:**

NCT06154473.

**Editor’s key points**

• This large multicentre prospective study examined early mortality and severe postoperative complications after cardiovascular surgery in Brazil.
• One-third of patients experienced early death or a major complication, with 78% of these events occurring on the first postoperative day.
• Independent risk factors included prior stroke, preoperative thrombocytopenia, open-heart surgery, biologic valve use, higher day-0 Sequential Organ Failure Assessment score, lower arterial pH on day 0, and postoperative vasoplegia.
• Commonly used risk scores, including ASA-Physical Status, Simplified Acute Physiology Score III, and European System for Cardiac Operative Risk Evaluation Score II, did not predict the primary outcome in multivariable analysis, highlighting the need for better-calibrated tools in low- and middle-income settings.
• The overall burden was substantial: 84% of patients required life support on day 0, and 79% developed at least one prespecified complication, underscoring the urgent need for locally relevant prognostic models to improve outcomes after cardiac surgery in Brazil.

Cardiac surgery remains a fundamental therapeutic modality in the management of cardiovascular disease, providing significant improvements in both survival and quality of life for appropriately selected patients.^1^^,^^2^ Open-heart and percutaneous surgery are now integral components of contemporary cardiovascular care.^3^^,^^4^ Globally, over 1 million cardiac surgical procedures are performed annually.^3^

Continuous advancements in surgical techniques, anaesthetic protocols, and perioperative management have contributed to improved outcomes, even among patients undergoing complex procedures.^5^, ^6^, ^7^, ^8^ However, cardiac surgery remains a high-risk, resource-intensive, and costly intervention, often associated with substantial perioperative morbidity and mortality.^3^^,^^9^ Beyond the intrinsic risks of the procedures themselves, patient outcomes are closely influenced by institutional expertise, surgical volume, and the availability of robust healthcare infrastructure.^10^^,^^11^

Cardiovascular disease is the leading cause of death worldwide, accounting for nearly 18 million deaths each year, more than 80% of which occur in low- and middle-income countries (LMICs).^3^ Despite this disproportionate burden, data on cardiac surgical outcomes in LMIC remain limited. This gap in knowledge underscores the urgent need for research aimed at characterising the epidemiology of patients undergoing cardiac surgery, intraoperative management, identifying perioperative risk factors, describe clinical outcomes, ultimately aiming to improve outcomes in these settings.

The Brazilian Surgical Identification Study 2 (BraSIS 2) aims to address this knowledge gap. We hypothesised that early mortality and severe postoperative complications are frequent among cardiac surgery patients in Brazil, and that specific preoperative, intraoperative, and postoperative factors are associated with these outcomes. Its primary aim is to assess the incidence of early mortality and severe postoperative complications in patients undergoing cardiovascular surgery in Brazil. In addition, the study aims to identify key risk factors for mortality or severe postoperative complications and to describe the patient characteristics and procedural characteristics of this patient population.

## Methods

### Study design and setting

This investigator-initiated, multicentre, prospective observational study was conducted across 11 centres in Brazil. The study protocol and statistical analysis plan were published and made publicly available before the completion of patient enrolment, as well as before data cleaning and database closure.^12^ During the inclusion period, all consecutive patients scheduled for cardiac surgery were screened for eligibility. The study start date at each participating centre was determined collaboratively with the central coordinating team and initiated only after approval by the respective local Institutional Review Board.

Ethical approval for this study (Ethical Committee N° CAAE: 69330823.1.0000.0071) was provided by the Ethical Committee of Hospital Israelita Albert Einstein, São Paulo, Brazil (Chairperson Prof Fabio P de S Santos) on 14 July 2023. Approval was also obtained from the Institutional Review Board of all participating institutions, in compliance with Brazilian regulatory requirements. The study was conducted and reported in accordance with the Strengthening the Reporting of Observational Studies in Epidemiology statement and the principles of Good Clinical Practice.^13^, ^14^, ^15^ The requirement for informed consent was determined by the Institutional Review Board at each site. When applicable, written informed consent was obtained from all participants. The study is registered on ClinicalTrials.gov (identifier: NCT06154473).

### Inclusion criteria

Adult patients (aged ≥18 yr) scheduled for cardiac surgery and anticipated to require postoperative care in the ICU were eligible for inclusion. Eligible surgical procedures included coronary artery bypass grafting (CABG), open-heart valve surgeries, including valvuloplasty and valve replacement, and percutaneous surgery. The percutaneous surgery included transcatheter aortic valve implantation (TAVI), valve-in-valve TAVI (ViV-TAVI), transcatheter mitral valve repair or replacement, and transcatheter bicaval valve implantation. Patients undergoing combined procedures, such as CABG with valve surgery performed during the same operative session, were also eligible for inclusion.

### Exclusion criteria

Patients were excluded if they were receiving exclusive palliative care before surgery, had advance directives limiting life support before surgery, had already been enrolled in the BraSIS 2 study, underwent surgery solely for the cardiac implantable electronic devices, or were deemed moribund preoperatively.

### Data collection and study variables

Study data were prospectively collected and managed using an electronic case report form implemented in the Research Electronic Data Capture (REDCap®; Vanderbilt University, Nashville, TN, USA) platform, hosted on secure servers at the Hospital Israelita Albert Einstein in São Paulo, Brazil.^16^^,^^17^ Data collection included variables from the preoperative, intraoperative, and postoperative periods.

To ensure data reliability, all investigators and research staff received standardised training on the identification and recording of predefined severe postoperative complications before study initiation. The electronic case report form included predefined validation rules, and a central coordinating team performed periodic consistency checks, cross-verification of key variables, and clarification queries with local investigators.^16^^,^^17^ In addition, random source document audits were conducted to confirm accuracy.

Collected variables included patient characteristics data (age, sex, race, weight, and height), comorbidities, type of surgery and anaesthesia, and relevant scores, such as the Sequential Organ Failure Assessment (SOFA) score,^18^ ASA Physical Status (ASA-PS) classification,^19^ Simplified Acute Physiology Score III (SAPS 3),^20^ and the EuroSCORE II.^21^ Mortality and postoperative complications were collected daily.

Additional data included the use of mechanical circulatory support devices, vasoactive drugs, mechanical ventilation,^22^ intraoperative complications,^23^ renal replacement therapy, laboratory blood test results, intraoperative monitoring and devices, transfusion of blood components and blood products, severe intraoperative complication, manoeuvres used to assess fluid responsiveness during the intraoperative period,^24^^,^^25^ daily fluid balance, and intravenous fluid administration.

Postoperative variables were recorded daily, starting from the day of admission to the ICU. The first time point (D0) covered data from ICU admission until 23:59 on the same day. Subsequent time points were recorded as D1, D2, and D3, corresponding to the first, second, and third full days after ICU admission. Patients were followed until ICU discharge, the end of D3, or death, whichever occurred first. All variables were collected as part of routine clinical care. A complete list of the data elements collected is available in the published study protocol.^12^

### Definition of severe postoperative complications

Severe postoperative complications were defined as the occurrence of any of the following adverse events: acute myocardial infarction,^26^ acute respiratory distress syndrome (ARDS) with P_a_O_2_/fraction of inspired oxygen ≤250 (refractory to rescue manoeuvres and ventilatory adjustment that persists for more than 1 h),^27^ cardiac arrest with return of spontaneous circulation, new surgical approach in an unscheduled manner of urgency or emergency, requirement for renal replacement therapy,^28^^,^^29^ septic shock,^30^ severe bleeding,^31^^,^^32^ severe haemodynamic instability,^33^^,^^34^ stroke, unplanned re-intubation, and unplanned use of a circulatory assistance device.^35^

Acute myocardial infarction was defined by the universal classification of type 5 infarction which is characterised by an elevation of troponin 10 times the 99th percentile associated by one of the following: development of new pathological Q waves; angiographic documented new graft occlusion or new native coronary artery occlusion; or imaging evidence of new loss of viable myocardium or new regional wall motion abnormality in a pattern consistent with an ischaemic aetiology.^26^ ARDS is defined according to the Berlin definition, excluding cases explained by heart failure or volume overload.^27^ Severe bleeding is defined as a drop of ≥2 g dl^−1^ of haemoglobin or transfusion of 2 units of packed red blood cells without increase in haemoglobin value or drop in systolic blood pressure ≥10 mm Hg upon standing, or spontaneous systolic blood pressure drop ≥20 mm Hg, or heart rate increase ≥20 beats min^−1^.^31^^,^^32^ Severe haemodynamic instability is defined as norepinephrine ≥0.1 μg kg^−1^ min^−1^ or epinephrine ≥0.1 μg kg^−1^ min^−1^ for more than 2 h; or their independent combination regardless of dose, or an independent combination of norepinephrine and vasopressin.^33^^,^^34^ Vasoplegia was defined as persistent vasodilatory shock despite adequate fluid resuscitation, occurring within the first 48 h after ICU admission and lasting for at least 2 consecutive hours with all of the following: mean arterial pressure ≤50 mm Hg and, when available, systemic vascular resistance ≤800 dyn·s cm^−5^; cardiac index ≥2.5 L min^−1^ m^−2^; and vasopressor requirements consistent with severe haemodynamic instability (as defined previously).^36^^,^^37^

### Primary outcome

The primary outcome was the incidence of mortality or severe postoperative complications occurring within the first 3 postoperative days or until ICU discharge, whichever occurs first. Patients discharged from the ICU after the postoperative day 3 without mortality or any of the predefined severe postoperative complications were classified as not having met the primary outcome.

### Secondary outcomes

Secondary outcomes were to describe risk factors for mortality or severe postoperative complications. Describe anaesthesia and surgical procedures. Assess the incidence of severe intraoperative complications such as bronchospasm or difficulties in respiratory support, extracorporeal circulation output failure (at least one failure of the first separation attempt or the need for circulatory assistance device to leave the operating room), cardiac arrest with return of spontaneous circulation, and excessive blood transfusion (>4 units of packed red cells). Assess the incidence of non-severe postoperative complications (complications not included in the primary outcome), such as cardiac tamponade, dysrhythmia (including atrial fibrillation, atrial flutter, sustained ventricular tachycardia, supraventricular tachycardia, and ventricular fibrillation), postoperative nausea and vomiting that is difficult to control, paralytic ileus, pneumonia, pneumothorax, psychomotor agitation (define as Richmond Agitation-Sedation Scale ≥+2),^38^ sustained diarrhoea, thrombocytopenia, unplanned need for oxygen, and vasoplegia. Describe patient characteristics and clinical profile of patients.

### Sample size

A minimum of 500 consecutive patients undergoing cardiac surgery were expected to be enrolled in the study. To enhance the representativeness of the findings across diverse clinical settings, each participating centre was limited to a maximum of 100 patients. There were no restrictions on the type of procedure, allowing for the inclusion of both open and percutaneous cardiac surgeries. Patients were enrolled consecutively to reflect routine clinical practice and to minimise selection bias, thereby ensuring that the study accurately captured standard approaches to cardiac surgical care across the participating institutions.

### Statistical analysis

A statistical analysis plan was published and made publicly available before the completion of patient enrolment, as well as before data cleaning and database closure.^12^ Patients were stratified according to the type of surgery, open-heart or percutaneous, and based on whether they experienced the primary outcome.

Baseline characteristics were summarised using counts and percentages for categorical variables, and as means with standard deviations or medians with interquartile ranges for continuous variables, depending on distribution. Comparisons between groups, such as type of surgery, were conducted using the chi-square test or Fisher’s exact test for categorical variables. Continuous variables were compared using the independent samples t-test or the Mann–Whitney U test, as appropriate based on normality assumptions. Between-centre variability was assessed using the intraclass correlation coefficient (ICC).

The incidence of the primary outcome was reported as absolute numbers and proportions and time until the first primary outcome was assessed using Kaplan–Meier survival curves. Associations between characteristics and the primary outcome were assessed using generalised linear mixed-effects models with a binomial distribution and an identity link function, accounting for the study centre as a random intercept to adjust for clustering. The model incorporated the centre as a random effect to account for potential heterogeneity between institutions. Results are presented as odds ratios (ORs) with corresponding 95% confidence intervals (CIs). To address scale issues in model fitting, pH values were multiplied by 10; thus, ORs for pH reflect a 0.1-unit increase.

Absolute risk differences (ARDs) with 95% CIs were estimated from the final model using marginal effects. Model performance was evaluated by discrimination, assessed with the area under the receiver-operating-characteristic curve (AUROC), and by calibration using the Hosmer–Lemeshow test. AUROC values were classified as excellent (≥0.9), very good (0.80–0.89), good (0.70–0.79), fair (0.60–0.69), or poor (<0.60).^39^

Relevant covariates included in the final multivariable model were identified as those with *P*<0.2 in the univariable model, clinical relevance, and no statistical association with other relevant variables. Minimal missing data were anticipated for exploratory outcomes. When missingness in core variables exceeded 10%, multiple imputation by chained equations was applied following standard procedures. For variables with ≤10% missing data, complete-case analysis was performed. All statistical tests were two-sided, with a significance threshold of *P*<0.05. No adjustments were made for multiple comparisons. All analyses were performed using R software, version 4.2.0 (R Core Team, Vienna, Austria).

## Results

### Patient and centre characteristics

Between September 2023 and March 2025, 11 hospitals in Brazil screened patients for inclusion in the BraSIS 2 study. Structural characteristics of participating centres, as well as the number of patients recruited at each site, are presented in Supplementary Table AF 1. Annual volumes of open-heart and percutaneous procedures by hospital are shown in Supplementary Tables AF 2–4.

Of the 556 eligible patients, 28 were excluded, resulting in a final cohort of 528 patients (Supplementary Fig. AF 1). During follow-up, 434 patients (82%) were discharged from the ICU, while 94 (18%) remained hospitalised. Among the 94 patients who were not discharged, 50 patients experienced at least one severe postoperative complication. No patients were lost to follow-up.

Baseline characteristics stratified by type of surgery are summarised in Table 1, and by the occurrence of mortality or severe postoperative complications in Supplementary Table AF 5. Open-heart surgery was performed in 479 patients (91%), whereas percutaneous procedures accounted for 49 cases (9%). Prespecified comorbidities were highly prevalent, affecting 495 of 528 patients (94%). The most frequent were hypertension (370 of 528, 70%), coronary artery disease (363 of 528, 69%), and diabetes mellitus (196 of 528, 37%). Notably, 342 patients (65%) presented with greater than or equal to three comorbidities. ASA-PS classification was most frequently III (355 of 528, 67%), underscoring the significant comorbidity burden in this cohort. Preoperative laboratory values were generally comparable between type of surgery, except for haemoglobin, which was higher among patients undergoing open-heart surgery (13.5 [standard deviation 1.8] *vs* 12.3 [2.0] g dl^−1^, *P*<0.001).

**Table 1:** Baseline characteristics of included patients, stratified by type of surgery (open *vs* percutaneous). Data are presented as mean [standard deviation] or as counts with percentages [*n* (%)]. *P*-values were calculated using the t-test for continuous variables and the chi-square test for categorical variables. Active endocarditis was defined as ongoing antibiotic therapy at the time of surgery. Heart failure was classified according to the left ventricular ejection fraction (LVEF): preserved function (LVEF ≥51%), intermediate function (LVEF 40–50%), reduced function (LVEF 30–39%), and very reduced function (LVEF ≤29%). Previous cardiac surgery was defined as any previous cardiac surgical procedure performed during a different hospitalisation. ASA-PS, ASA Physical Status; CKD, chronic kidney disease; COPD, chronic obstructive pulmonary disease; EUROSCORE, European System for Cardiac Operative Risk Evaluation; Hb, haemoglobin; LVEF, left ventricular ejection fraction; RRT, renal replacement therapy; SAPS, Simplified Acute Physiology Score; SOFA, Sequential Organ Failure Assessment.

	All patients (*n=*528)	Open-heart (*n=*479)	Percutaneous (*n=*49)
Age, yr	22-95	22-90	54-95
BMI (kg m^−2^)	27 [5]	27 [5]	25 [3]
Male sex	377 (71%)	352 (73%)	25 (51%)
ASA-PS classification			
ASA-2	109 (21%)	99 (21%)	10 (20%)
ASA-3	355 (67%)	327 (68%)	28 (57%)
ASA-4	64 (12%)	53 (11%)	11 (22%)
EUROSCORE II	2 [3]	2 [3]	4 [5]
SOFA score D0	4 [3]	5 [2]	2 [2]
SAPS 3 score	42 [12]	41 [12]	45 [11]
Comorbidities	495 (94%)	448 (94%)	47 (96%)
Hypertension	370 (70%)	333 (70%)	37 (76%)
Coronary artery disease	363 (69%)	339 (71%)	24 (49%)
Diabetes mellitus	196 (37%)	176 (37%)	20 (40%)
Heart failure	171 (32%)	148 (31%)	23 (46%)
Preserved function	82 (6%)	73 (15%)	9 (18%)
Intermediate function	49 (6%)	47 (10%)	2 (4%)
Reduced function	25 (6%)	18 (4%)	7 (14%)
Very reduced function	15 (6%)	10 (2%)	5 (10%)
Unstable angina <90 days	125 (24%)	120 (25%)	5 (10%)
Thrombocytopenia	83 (16%)	68 (14%)	15 (30%)
Atrial fibrillation or flutter	56 (11%)	49 (10%)	7 (14%)
Previous heart surgery	53 (10%)	47 (10%)	6 (12%)
Stroke	31 (6%)	26 (5%)	5 (10%)
CKD not requiring RRT	30 (6%)	21 (4%)	9 (18%)
CKD requiring RRT	10 (2%)	10 (2%)	0 (0%)
COPD	29 (5%)	25 (5%)	4 (8%)
Anaemia (Hb <10 g dl^−1^)	23 (4%)	21 (4%)	2 (4%)
Obstructive sleep apnoea	23 (4%)	21 (4%)	2 (4%)
Rheumatic fever	19 (4%)	18 (4%)	1 (2%)
Asthma	14 (3%)	12 (3%)	2 (4%)
Active endocarditis	12 (2%)	12 (3%)	0 (0%)
Active cancer	9 (2%)	6 (1%)	3 (6%)
Laboratory tests before surgery			
Creatinine (mg dl^−1^)	1.16 [0.6]	1.15 [0.7]	1.22 [0.4]
Glucose (mg dl^−1^)	126 [63]	127 [65]	112 [26]
Haemoglobin (g dl^−1^)	13.3 [1.8]	13.5 [1.8]	12.3 [2.0]
Ionic calcium (mmolL^−1^)	1.18 [0.1]	1.18 [0.1]	1.15 [0.1]

Compared with patients undergoing percutaneous procedures, those undergoing open-heart surgery were significantly younger (62 [11] *vs* 79 [9] yr, *P*<0.001), more frequently male (352 [73%] *vs* 25 [51%], *P*=0.002), and presented with lower EuroSCORE II (2 [3] *vs* 4 [5], *P*=0.021) and lower SAPS 3 scores on postoperative day 0 (41 [12] *vs* 45 [11], *P*=0.011). In contrast, SOFA scores on postoperative day 0 were higher in open-heart surgery patients (5 [2] *vs* 2 [2], *P*<0.001).

### Primary outcome

Mortality or severe postoperative complications occurred in 170 patients (32%) within the first 3 postoperative days (Table 2). The primary outcome was more frequent among patients undergoing open-heart surgery than among those undergoing percutaneous procedures (163 [34%] *vs* seven [14%]). ICU mortality was observed in 15 patients (3%). All patients who died had also experienced at least one prespecified severe postoperative complication. Given the low number of deaths, the observed associations are driven predominantly by severe postoperative complications rather than mortality. Accordingly, these findings should not be interpreted as evidence that the identified factors are predictors of mortality. The most frequent severe postoperative complications were severe haemodynamic instability in 126 patients (24%), followed by ARDS in 71 patients (13%), and severe bleeding in 33 patients (6%). Kaplan–Meier estimates for the composite outcome of mortality or severe postoperative complications are shown in Supplementary Fig. AF 2. Most events occurred on the day of ICU admission (132 [78%] of 170), with a marked decline thereafter: 27 events (16%) on day 1, eight (5%) on day 2, and three (2%) on day 3. Substantial variability was observed across centres. The incidence of the primary outcome ranged from 10% to 56%, whereas ICU mortality ranged from 0% to 7%. The ICC was 0.26. No clear association between surgical volume and adverse outcomes was identified (Supplementary Table AF 4).

**Table 2:** Clinical outcomes of included patients, stratified by type of surgery (open *vs* percutaneous). Data are presented as mean [standard deviation] or as counts with percentages [*n* (%)]. Dysrhythmia was defined as the occurrence of any of the after atrial fibrillation, atrial flutter, sustained ventricular tachycardia, or supraventricular tachycardia. Thrombocytopenia was defined as platelet count of <150 000 mm^3^. For the individual components, a patient can be scored for more than one complication. As consequence, the sum of the numbers of each individual component will not result in the total number of intraoperative or postoperative complications. ARDS, acute respiratory distress syndrome.

	All patients (*n=*528)	Open-heart (*n=*479)	Percutaneous (*n=*49)
**Primary outcomes**			
ICU mortality or severe postoperative complications	170 (32%)	163 (34%)	7 (14%)
ICU mortality	15 (3%)	14 (3%)	1 (2%)
Severe postoperative complications	170 (32%)	163 (34%)	7 (14%)
Severe haemodynamic instability	126 (24%)	121 (25%)	5 (10%)
ARDS	71 (13%)	70 (15%)	1 (2%)
Severe bleeding	33 (6%)	32 (7%)	1 (2%)
Unplanned reintubation	15 (3%)	15 (3%)	0 (0%)
Unplanned new surgical approach	14 (3%)	12 (3%)	2 (4%)
Renal replacement therapy	8 (2%)	8 (2%)	0 (0%)
Septic shock	8 (2%)	8 (2%)	0 (0%)
Unplanned use of a circulatory assistance device	4 (1%)	4 (1%)	0 (0%)
Stroke	4 (1%)	4 (1%)	0 (0%)
Acute myocardial infarction	3 (1%)	3 (1%)	0 (0%)
Cardiorespiratory arrest with return of spontaneous circulation	2 (0%)	2 (0%)	0 (0%)
**Secondary outcomes**			
Severe intraoperative complications	33 (6%)	33 (7%)	0 (0%)
Extracorporeal circulation output failure	16 (3%)	16 (3%)	0 (0%)
Excessive blood transfusion	11 (2%)	11 (2%)	0 (0%)
Cardiac arrest with return of spontaneous circulation	4 (1%)	4 (1%)	0 (0%)
Bronchospasm or difficulties in respiratory support	1 (0%)	1 (0%)	0 (0%)
Postoperative complications	388 (73%)	356 (74%)	32 (65%)
Thrombocytopenia	345 (65%)	315 (66%)	30 (61%)
Dysrhythmia	64 (12%)	60 (13%)	4 (8%)
Vasoplegia	59 (11%)	53 (11%)	6 (12%)
Unplanned need for oxygen supplementation	46 (9%)	43 (9%)	3 (6%)
Psychomotor agitation	24 (5%)	22 (5%)	2 (4%)
Postoperative nausea and vomiting	16 (3%)	15 (3%)	1 (2%)
Pneumonia	10 (2%)	10 (2%)	0 (0%)
Paralytic ileus	6 (1%)	6 (1%)	0 (0%)
Sustained diarrhoea	3 (1%)	3 (1%)	0 (0%)
Cardiac tamponade	1 (0%)	1 (0%)	0 (0%)
Pneumothorax	0 (0%)	0 (0%)	0 (0%)

### Secondary outcomes

#### Risk factors for mortality or severe postoperative complications

**Fig 1:**
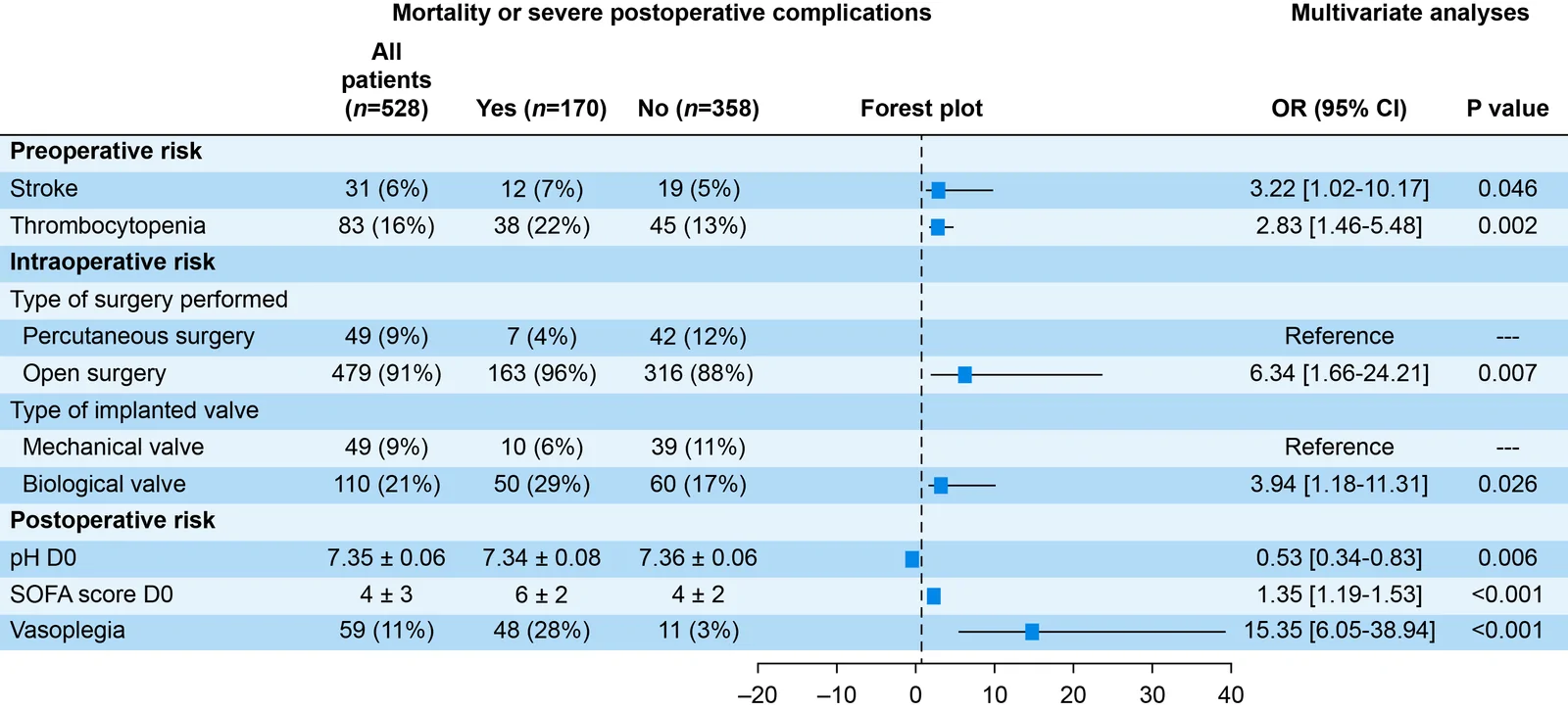
Factors related to the risk of mortality or severe postoperative complications. CI, confidence interval; OR, odds ratio; SOFA, Sequential Organ Failure Assessment.

Several factors were significantly associated with mortality or severe postoperative complications (Fig. 1). Covariates included in univariate and multivariable models are detailed in Supplementary Table AF 6, and variables requiring multiple imputation are shown in Supplementary Table AF 7. The model demonstrated very good discrimination, with an AUROC of 0.888 (95% CI, 0.860–0.916), and adequate calibration, as assessed by the Hosmer–Lemeshow test (X^2^=7.690, *P*=0.464). These performance metrics should be interpreted as applicable only to the present cohort and outcome definition.

Preoperative risk factors included previous stroke (OR 3.22 [1.02–10.17], *P*=0.046; and ARD 0.19 [0.00–0.39]) and preoperative thrombocytopenia (OR 2.83 [1.46–5.48] *P*=0.002; and ARD 0.12 [0.02–0.21]). Intraoperatively, open-heart surgery (OR 6.34 [1.66–24.21], *P*=0.007; and ARD 0.18 [0.07–0.29]), and the use biological valves (OR 3.94 [1.18–11.31], *P*=0.026; and ARD 0.14 [0.03–0.26]) were associated with higher risk. Postoperative risk factors included higher SOFA scores on day 0 (OR 1.35 [1.19–1.53], *P*<0.001; and ARD 0.04 [0.02–0.06]), lower arterial pH on day 0 (OR 0.53 [0.34–0.83], *P*=0.006; and ARD −0.09 [−0.14 to −0.04]) and development of vasoplegia (OR 15.35 [6.05–38.94], *P*<0.001; and ARD 0.45 [0.31–0.60]).

#### Anaesthesia and surgical procedures

General anaesthesia was the predominant anaesthetic technique, administered to 338 patients (64%), followed by combined anaesthesia in 176 (33%). Sedation was performed exclusively in percutaneous procedures; however, even in this subgroup, general anaesthesia remained the most frequent approach. Intraoperative data are summarised by surgical type in Table 3 and by occurrence of mortality or severe postoperative complications in Supplementary Table AF 8.

**Table 3:** Intraoperative data of included patients, stratified by type of surgery (open *vs* percutaneous). Data are presented as mean [standard deviation] or as counts with percentages [*n* (%)]. *P*-values were calculated using the t-test for continuous variables and the chi-square test for categorical variables.

	All patients (*n=*528)	Open-heart (*n=*479)	Percutaneous (*n=*49)
**Anaesthesia administered**			
General anaesthesia	338 (64%)	309 (65%)	29 (59%)
Combined anaesthesia	176 (33%)	170 (35%)	6 (12%)
Sedation	14 (3%)	0 (0%)	14 (29%)
**Complications during anaesthesia**	33 (6%)	33 (7%)	0 (0%)
**Duration of surgery, min**	221 [91]	227 [89]	151 [87]
**Total fluid intake (ml)**	2652 [1,638]	2753 [1,639]	1281 [774]
**Transfusion of blood products**	118 (22%)	116 (24%)	2 (4.1%)
Red blood cells	95 (18%)	95 (20%)	0 (0%)
Platelets	38 (7%)	36 (8%)	2 (4%)
Fresh frozen plasma	13 (2%)	13 (3%)	0 (0%)
Cryoprecipitate	12 (2%)	12 (3%)	0 (0%)
**Type of surgery performed**			
Elective	430 (81%)	391 (82%)	39 (80%)
Urgent/emergency	98 (19%)	88 (18%)	10 (20%)
**Urine output (ml)**	1315 [1770]	1380 [1809]	398 [527]

The most common procedure overall was CABG in 322 patients (61%), followed by valve replacement in 161 patients (30%) (Table 4). Among percutaneous surgeries, TAVI was the most frequently performed procedure in 43 patients (8%) (Table 4). Among patients undergoing open-heart surgery, extracorporeal circulation was employed in 424 of 479 cases (89%), and in CABG patients, the mean number of grafts was 2.5. In open-heart valve replacement surgeries, biological valve was more frequently adopted than mechanical valve (110 [21%] *vs* 49 [9%]) (Table 4). Support interventions, medications, and intraoperative devices are detailed in Supplementary Table AF 9.

**Table 4:** Type of surgery performed stratified according to the occurrence of mortality or severe postoperative complications. Note: Data are presented as mean [standard deviation] or as counts with percentages [*n* (%)]. CABG, coronary artery bypass grafting; TAVI, transcatheter aortic valve implantation; ViV-TAVI, valve-in-valve TAVI.

	All patients (*n=*528)	Yes (*n=*170)	No (*n=*358)
**Open surgery**	479 (91%)	163 (96%)	316 (88%)
**CABG**	322 (61%)	114 (67%)	208 (58%)
Number of grafts performed	2.5 [1.0]	2.5 [0.8]	2.6 [1.0]
Extracorporeal circulation	424 (80%)	144 (85%)	280 (78%)
Cardiopulmonary bypass time, min	79 [35]	88 [37]	75 [34]
Aortic cross-clamping time, min	59 [28]	65 [30]	56 [27]
**Valve replacement**	161 (30%)	61 (36%)	100 (28%)
Aortic valve	102 (19%)	35 (21%)	67 (19%)
Mitral valve	69 (13%)	32 (19%)	37 (10%)
Tricuspid valve	1 (0%)	0 (0%)	1 (0%)
**Valvuloplasty**	45 (9%)	18 (11%)	27 (8%)
Mitral valve	29 (5%)	10 (6%)	19 (5%)
Aortic valve	10 (2%)	5 (3%)	5 (1%)
Tricuspid valve	9 (2%)	5 (3%)	4 (1%)
**Type of implanted valve**	159 (30%)	60 (35%)	99 (28%)
Biological	110 (21%)	50 (29%)	60 (17%)
Mechanical	49 (9%)	10 (6%)	39 (11%)
**Percutaneous surgery**	49 (9%)	7 (4%)	42 (12%)
TAVI	43 (8%)	6 (4%)	37 (10%)
Transcatheter treatment of the mitral valve	4 (1%)	0 (0%)	4 (1%)
ViV-TAVI	2 (0%)	1 (1%)	1 (0%)

#### Severe intraoperative complications

Prespecified severe intraoperative complications occurred in 33 patients (6%), all of whom underwent open-heart surgery. No severe intraoperative complications were reported in percutaneous surgeries. The most frequent complications were extracorporeal circulation output failure in 16 patients (3%) and excessive blood transfusion in 11 patients (2%) (Table 2).

#### Non-severe postoperative complications

Non-severe postoperative complications occurred in 388 patients (73%) (Table 2). The most frequent were thrombocytopenia in 345 patients (65%), followed by cardiac dysrhythmias in 64 patients (12%), vasoplegia in 59 patients (11%), and unplanned oxygen supplementation in 46 patients (9%). When considering all postoperative complications (include in the primary or in the secondary outcomes), 415 patients (79%) experienced at least one prespecified complication.

#### Life-support interventions

On postoperative day 0, 445 patients (84%) required life-support interventions (Supplementary Table AF 10). Of these, vasoactive drugs were administered to 392 patients (74%), most frequently norepinephrine in 270 patients (51%), followed by dobutamine in 201 patients (38%). Invasive mechanical ventilation was required in 297 patients (56%). Renal replacement therapy was used in four cases, intra-aortic balloon pump in three, and extracorporeal membrane oxygenation in one. The need for life support declined markedly over time, from 445 patients (84%) on day 0, 339 patients (65%) on day 1, 212 patients (44%) on day 2, and 127 patients (38%) on day 3 (Supplementary Table AF 10 and Table AF 11).

## Discussion

In this prospective multicentre cohort of 528 patients undergoing cardiac surgery in Brazil, one in three developed early mortality or severe postoperative complications, most within the first 24 h. In the immediate postoperative phase, severe haemodynamic instability and ARDS were the most frequent complications. Preoperative and intraoperative predictors included previous stroke, preoperative thrombocytopenia, open-heart surgery, and biological valve use, whereas postoperative variables, such as higher SOFA scores, lower arterial pH, and postoperative vasoplegia, should be interpreted as early markers of clinical deterioration rather than baseline predictors of risk. Given the relatively low number of deaths, the observed associations are driven predominantly by severe postoperative complications rather than mortality; accordingly, these findings should not be interpreted as evidence that the identified factors are independent predictors of mortality.

Severe postoperative complications occurred in 32% of patients, while additional complications affected 73%. Overall, 79% of patients experienced at least one prespecified complication. These rates are consistent with previous cohorts, such as a study of 26 221 patients undergoing CABG and/or valve surgery that reported complications in 66.6% with survival declining as complications accumulated.^40^ In our study, 84% required life support on day 0, underscoring the clinical complexity, resource demands, and economic burden of this population.

The high rate of events on ICU admission day likely reflects perioperative physiological stress, including baseline vulnerability, surgical insult, and vasoplegia.^36^^,^^37^ Importantly, postoperative variables (SOFA D0, pH D0, vasoplegia D0) should be interpreted as early markers of deterioration rather than baseline predictors, supporting their use for dynamic risk stratification.^41^^,^^42^ Beyond individual-level risk factors, these findings have important implications for healthcare delivery. Substantial between-centre variability suggests a meaningful institutional effect.^41^^,^^42^ Although no clear association between surgical volume and outcomes was observed, this may reflect the predominance of academic centres and shared surgical teams across hospitals, limiting volume as a proxy for expertise. Collectively, these findings suggest that improving outcomes in cardiac surgery requires not only optimisation of individual patient care but also the adoption of system-level strategies, including regionalisation of services, protocol standardisation, and context-specific refinement of risk prediction models across the perioperative continuum. However, given the pragmatic nature of data collection, it is not possible to determine whether day 0 complications are primarily driven by patient-related vulnerability, surgical factors, or vasoplegic phenomena.

Preoperative stroke was present in 6% of patients, while postoperative stroke occurred in 1%, exclusively after open-heart surgery, consistent with previous report.^43^ Previous stroke predicted adverse outcomes, reinforcing the well-established perioperative vulnerability of this population. Although we did not assess the interval between stroke and surgery, previous research shows markedly higher risks when procedures are performed soon after an ischaemic event.^43^

Preoperative thrombocytopenia was observed in 16% of patients, while postoperative thrombocytopenia developed in 65%. This incidence is substantially higher than previously reported in the literature, where rates of postoperative thrombocytopenia are closer to 26%, and its occurrence has been consistently associated with increased mortality and postoperative complications.^44^ Given the critical role of platelets in haemostasis and organ function, reduced platelet counts are a well-recognised marker of poor prognosis.^18^^,^^44^ Our study provides evidence that preoperative thrombocytopenia may predicts mortality and severe postoperative complications.

Percutaneous surgery was associated with a lower risk of adverse outcomes compared with open-heart surgery. Nevertheless, surgical decision-making should remain guided by a multidisciplinary heart team, as selected patients may still benefit from open-heart surgery.^1^^,^^2^^,^^45^^,^^46^ Patients referred for open surgery often represent a higher-risk group, which may contribute to less favourable outcomes.^1^ In our cohort, however, patients undergoing percutaneous surgery presented with higher baseline risk, as evidenced by older age, higher EuroSCORE II, and greater illness severity at ICU admission (higher SAPS 3); yet they experienced a more favourable early postoperative course, with lower SOFA scores on day 0, when most adverse events occurred. These findings support prioritising percutaneous surgery when both strategies are clinically appropriate.

Mechanical valve implantation was associated with a lower risk of adverse outcomes compared with biological valves. Nevertheless, biological valves were implanted more than twice as often in our cohort, likely reflecting institutional practice patterns and patient selection. The optimal prosthesis remains debated, particularly regarding the balance between anticoagulation risks, reoperation rates, and patient age.^47^, ^48^, ^49^ A retrospective analysis of 324 patients reported higher bleeding risk with mechanical prostheses, whereas biological valves offered a short-term survival advantage that disappeared after 10 yr of follow-up.^47^ In contrast, a study of 2056 patients demonstrated superior long-term survival with mechanical prostheses, with age significantly influencing outcomes.^48^ Similarly, in a cohort of 1443 patients, mechanical prostheses were associated with higher rates of major bleeding, whereas bioprostheses were linked to more frequent reoperations; in this context, implantation of a bioprostheses in patients older than 55 yr was considered reasonable.^49^ Although our study did not evaluate long-term follow-up, it adds evidence suggesting that mechanical valves confer a short-term protective effect.

Higher SOFA scores^18^ on postoperative day 0 were associated with worse outcome. Although this variable is not modifiable, it highlights patients at particularly high risk who require closer monitoring and frequent reassessment. In contrast, traditional scores such as ASA physical status,^19^ SAPS 3,^20^ and EuroSCORE II,^21^ were not associated with adverse outcomes within the early postoperative window analysed, suggesting limited predictive accuracy in this Brazilian cohort. Furthermore, the observed heterogeneity in outcomes underscores the limitations of traditional preoperative risk scores. It is unlikely that a single risk model can achieve optimal predictive performance across diverse populations and healthcare systems.^39^ The observed lack of association may therefore reflect the absence of LMIC populations in their original development. Many existing tools emphasise comorbidities and disease patterns predominant in high-income countries, which may not fully capture risks in resource-limited contexts.^50^^,^^51^ Importantly, multidisciplinary heart teams in LMIC should exercise caution when applying universal scores not validated in their context, recognising their potential limitations for decision-making.

Lower arterial pH on postoperative day 0 was also associated with worse outcome. The clinical applicability of this finding remains uncertain. Low pH may primarily reflect disease severity rather than a modifiable therapeutic target; nonetheless, it serves as a useful early marker to identify patients at high risk in the immediate postoperative period. Although interventions such as intravenous bicarbonate or renal replacement therapy can increase pH, their effect on outcomes is unclear, and these strategies carry inherent risks.^28^^,^^29^^,^^52^ Our results therefore suggest that patients presenting with lower arterial pH should be recognised as high risk, warranting closer monitoring and more frequent reassessment, particularly in the early postoperative phase.

The development of vasoplegia was associated with mortality and severe complications. However, it should be acknowledged that no universally accepted definition of vasoplegia currently exists, and variability in diagnostic criteria may substantially influence reported incidence rates and associated outcomes.^36^^,^^37^ Vasoplegia occurs in approximately 10%–20% of patients after CABG; however, preventive strategies remain uncertain.^4^ Early recognition is crucial, and vasoplegic syndrome should be suspected in patients with hypotension despite a normal or elevated cardiac index.^4^ First-line therapy typically involves norepinephrine. Vasopressin (0.01–0.04 U min^−1^) may be added, as studies have shown it can improve haemodynamic stability and reduce progression to distributive shock.^4^

The BraSIS 2 study has several strengths. First, it was a multicentre investigation that reflected clinical practice across multiple hospitals, thereby increasing the generalisability of the findings. Second, data were collected within a relatively short time window, minimising the influence of temporal changes in practice. Third, outcomes and analytic methods were prespecified, and both the study protocol and the statistical analysis plan had been published beforehand, ensuring transparency and methodological rigour. Finally, the prospective design allowed for systematic collection of perioperative, intraoperative, and postoperative data, enabling robust identification of risk factors associated with complications and mortality in patients undergoing cardiac surgery.

Several limitations should be acknowledged. First, data collection in the BraSIS 2 study was restricted to routine clinical practice, without additional laboratory or radiological assessments beyond standard care. As a result, some postoperative complications, particularly neurological or subclinical events, may have gone undetected. Second, potential sources of bias must be considered. For example, anaesthesiologists, surgeons, and intensivists may have altered their practice owing to awareness of being observed (Hawthorne effect). Third, the follow-up period was limited to 3 postoperative days or until ICU discharge, whichever occurred first; therefore, complications or readmissions occurring beyond this timeframe were not captured. Fourth, because of the pragmatic design, intraoperative data and less frequent complications were collected in a simplified manner to maximise feasibility and adherence across participating centres. This approach entails an inherent trade-off between feasibility and data granularity, which may limit mechanistic interpretation. Fifth, although we intended to perform separate models for mortality and for severe postoperative complications, the small number of deaths (*n=*15, 3%) limited the statistical robustness of such analyses. In addition, because the study was designed to reflect real-world practice, procedural indications and treatment allocation (open-heart *vs* percutaneous) were not standardised or controlled. Consequently, selection bias and confounding by indication may have influenced these comparisons, and the study design does not permit determination of whether observed differences between procedural groups were attributable to the procedures themselves or to underlying differences in baseline patient characteristics. The limited sample size constrained the robustness of these analyses; accordingly, they should be considered exploratory and hypothesis-generating rather than confirmatory. Finally, the observational design precludes causal inference; thus, the reported associations should be interpreted as hypothesis-generating and require confirmation in future prospective, controlled studies.

These results have important implications for clinical practice in Brazil and potentially in other LMIC, although generalisation to other healthcare systems should be made with caution, as all participating centres are part of single national healthcare system. Early recognition of high-risk patients, particularly those with previous stroke, preoperative thrombocytopenia, or postoperative vasoplegia, may allow for targeted monitoring and timely interventions. The findings also underscore the need for multicentre studies with longer follow-up to evaluate late outcomes and for the development of prognostic models validated in local contexts.^50^^,^^51^

### Conclusion

In this large multicentre cohort of cardiac surgery patients in Brazil, approximately one in three experienced early mortality or severe postoperative complications, mostly within the first 24 h. Identified predictors of early postoperative deterioration may help guide perioperative strategies and underscore the need for developing prognostic models adapted to local contexts.

## Authors’ contributions

Conceived the study: RCFC, NNBV, TDC, JMSJ

Wrote the initial manuscript: RCFC

Revised the manuscript: all authors

Enrolled the study participants: all authors

Collected the data: all authors

Analysed the data: RCFC, TMS

Approved the final version of the manuscript: all authors

## Supplementary material

Supplementary material is available at *British Journal of Anaesthesia* online.

## Presentation

Preliminary data from this study have not been previously presented.

## Data availability statement

The data underlying this study may be made available upon reasonable request. Access to the de-identified dataset can be granted after evaluation of a research proposal. Requests for data access should be directed to the corresponding author (renato.carneiro@einstein.br).

## Declaration of interest

The authors declare that they have no conflicts of interest.
