# Supplementary material for: Epidemiology, clinical outcomes and risk factors for mortality and severe postoperative complications in cardiac surgery patients admitted to intensive care units in Brazil: a multicentre observational study
Source: BJA Open. 2026 Aug 12;19:100580. doi: 10.1016/j.bjao.2026.100580 (PMC13499056; doi:10.1016/j.bjao.2026.100580)
Supplement: Supplementary file 1 [file mmc1.docx]

Additional File

**Figure AF 1.** Study flow chart.

**Legend of figure AF 1.** Note: ^A^ Non-eligible surgical procedure included the following: 11 patients who underwent surgery exclusively for the implantation of cardiac implantable electronic devices, 2 patients who underwent resection of atrial tumours, and 2 patients who underwent repair of an ostium secundum-type atrial septal defect.

**Figure AF 2.** Kaplan–Meier estimates for the composite outcome of mortality or severe postoperative complications.

**RESULTS**

**Table AF1.** Structural characteristics of hospitals.

| **Hospital** | **ICU Type** | **Hospital Ownership** | **Academic Status** | **ICU residents or fellows** | **Cardiac Surgery residents or fellows** | **Anaesthesiology residents or fellows** | **Adult ICU beds (n)** | **Hospital beds (n)** | **Intermediate care unit beds** | **Number included patients** |
| --- | --- | --- | --- | --- | --- | --- | --- | --- | --- | --- |
| Hospital 1 | Cardiologic | Mixed public–private | Teaching or university | Yes | Yes | Yes | 40 | 130 | 0 | 100 |
| Hospital 2 | Mixed clinical–surgical | Public-private | Teaching or university | Yes | No | Yes | 40 | 317 | 10 | 87 |
| Hospital 3 | General | Private | Teaching or university | Yes | No | Yes | 69 | 681 | 85 | 69 |
| Hospital 4 | Cardiologic | Public | Teaching or university | Yes | Yes | Yes | 168 | 465 | 0 | 63 |
| Hospital 5 | Mixed clinical–surgical | Private | Teaching or university | Yes | Yes | No | 73 | 310 | 0 | 53 |
| Hospital 6 | General | Public | Teaching or university | Yes | Yes | Yes | 66 | 1000 | 7 | 44 |
| Hospital 7 | General | Public | Teaching or university | Yes | Yes | Yes | 84 | 584 | 0 | 41 |
| Hospital 8 | Cardiologic | Public | Teaching or university | Yes | No | Yes | 30 | 420 | 18 | 35 |
| Hospital 9 | General | Private | Community | No | No | No | 30 | 123 | 10 | 20 |
| Hospital 10 | Cardiologic | Private | Community | Yes | No | No | 5 | 280 | 0 | 15 |

Note: data from one centre were not included in this table, as it recruited only one patient.

**Table AF2.** Annual volume of open-heart surgical procedures by hospital.

| **Hospital** | 2023 | | | | 2024 | | | | 2025 | | | |
| --- | --- | --- | --- | --- | --- | --- | --- | --- | --- | --- | --- | --- |
|  | **CABG** | **valvuloplasty** | **valve replacement** | **Combined procedures** | **CABG** | **valvuloplasty** | **valve replacement** | **Combined procedures** | **CABG** | **valvuloplasty** | **valve replacement** | **Combined procedures** |
| Hospital 11 | 135 | 2 | 55 | 19 | 154 | 0 | 56 | 16 | 144 | 0 | 54 | 16 |
| Hospital 12 | 101 | 5 | 43 | 0 | 102 | 10 | 29 | 0 | 100 | 10 | 36 | 0 |
| Hospital 13 | 624 | 2 | 266 | 1 | 524 | 2 | 285 | 4 | 569 | 11 | 292 | 6 |
| Hospital 14 | 187 | 118 | 39 | 129 | 169 | 113 | 61 | 118 | 150 | 112 | 51 | 125 |
| Hospital 15 | 76 | 2 | 34 | 2 | 73 | 12 | 39 | 1 | 78 | 7 | 47 | 6 |
| Hospital 16 | 84 | 35 | 63 | 0 | 84 | 40 | 82 | 0 | 73 | 24 | 79 | 0 |
| Hospital 17 | 97 | 5 | 26 | 5 | 79 | 4 | 27 | 3 | 63 | 3 | 15 | 4 |
| Hospital 18 | 27 | 4 | 18 | 3 | 25 | 10 | 13 | 6 | 21 | 15 | 32 | 2 |
| Hospital 19 | 73 | 0 | 18 | 3 | 75 | 2 | 12 | 6 | 62 | 8 | 13 | 7 |
| Hospital 20 | 27 | 9 | 28 | 3 | 24 | 15 | 43 | 3 | 33 | 11 | 31 | 0 |

Note: Hospitals included in this Table are coded from 11 to 20 to distinguish them from those presented in Table AF1 (coded 1–10). This renumbering was performed to preserve institutional anonymity and to prevent potential identification or benchmarking across centres.

Combined procedures include CABG plus valve surgery or other open-heart procedures performed during the same operative session. Data from one centre were not included in this Table, as it recruited only one patient.

**Table AF3.** Annual volume of percutaneous procedures by hospital.

| **Hospital** | **2023** | | **2024** | | **2025** | |
| --- | --- | --- | --- | --- | --- | --- |
|  | **TAVI or ViV-TAVI** | **Transcatheter mitral or bicaval Implantation** | **TAVI or ViV-TAVI** | **Transcatheter mitral or bicaval Implantation** | **TAVI or ViV-TAVI** | **Transcatheter mitral or bicaval Implantation** |
| Hospital 11 | 12 | 0 | 9 | 0 | 13 | 0 |
| Hospital 12 | 16 | 0 | 9 | 0 | 5 | 0 |
| Hospital 13 | 38 | 1 | 28 |  | 105 | 0 |
| Hospital 14 | 71 | 7 | 77 | 7 | 85 | 11 |
| Hospital 15 | 0 | 0 | 6 | 0 | 31 | 0 |
| Hospital 16 | 57 | 8 | 49 | 5 | 49 | 8 |
| Hospital 17 | 0 | 0 | 8 | 0 | 3 | 0 |
| Hospital 18 | 0 | 0 | 16 | 0 | 25 | 0 |
| Hospital 19 | 123 | 0 | 143 | 0 | 117 | 0 |
| Hospital 20 | 10 | 4 | 17 | 4 | 15 | 5 |

Note: Hospitals included in this Table are coded from 11 to 20 to distinguish them from those presented in Table AF1 (coded 1–10). This renumbering was performed to preserve institutional anonymity and to prevent potential identification or benchmarking across centres. Transcatheter mitral procedures and bicaval valve implantation, as well as transcatheter aortic valve implantation (TAVI) and valve-in-valve TAVI (ViV-TAVI), are presented as aggregated categories. Given the small number of cases across centres, this approach allows reporting of procedural volumes while minimising the risk of centre identification. Data from one centre were not included in this table, as it recruited only one patient.

**Table AF4.** Annual volume of open-heart surgical procedures and proportion of the primary outcome by hospital

| **Hospital** | **Open-Heart Surgeries** | | | **Percutaneous Cardiac Procedures** | | | **Severe postoperative complications** | **ICU mortality** |
| --- | --- | --- | --- | --- | --- | --- | --- | --- |
|  | **2023** | **2024** | **2025** | **2023** | **2024** | **2025** |  |  |
| Hospital 11 | 211 | 226 | 214 | 12 | 9 | 13 | 56% | 3% |
| Hospital 12 | 149 | 141 | 145 | 16 | 9 | 5 | 52% | 2% |
| Hospital 13 | 893 | 815 | 878 | 39 | 28 | 105 | 35% | 3% |
| Hospital 14 | 473 | 461 | 438 | 78 | 84 | 96 | 30% | 0% |
| Hospital 15 | 114 | 125 | 138 | 0 | 6 | 31 | 24% | 2% |
| Hospital 16 | 182 | 206 | 176 | 65 | 54 | 57 | 22% | 0% |
| Hospital 17 | 133 | 113 | 85 | 0 | 8 | 3 | 20% | 6% |
| Hospital 18 | 52 | 54 | 70 | 0 | 16 | 25 | 20% | 6% |
| Hospital 19 | 94 | 95 | 90 | 123 | 143 | 117 | 13% | 7% |
| Hospital 20 | 67 | 85 | 75 | 14 | 21 | 20 | 10% | 0% |

Note: Hospitals included in this Table are coded from 11 to 20 to distinguish them from those presented in Table AF1 (coded 1–10). This renumbering was performed to preserve institutional anonymity and to prevent potential identification or benchmarking across centres. Data from one centre were not included in this table, as it recruited only one patient. This Table presents only the proportion of events per centre, to not inform the number of patients included, that could potentially allow the identification of the centre.

**Table AF 5.** Baseline characteristics of included patients, stratified by the occurrence of mortality or severe postoperative complications.

|  | **All patients**  **(*n=*528)** | **Yes**  **(*n=*170)** | **No**  **(*n=*358)** | **P**  **value** |
| --- | --- | --- | --- | --- |
| Age, yr | 64 [12] | 66 [11] | 63 [13] | 0.006 |
| BMI (kg/m^2^) | 27 [5] | 27 [4] | 28 [5] | 0.103 |
| Male sex | 377 (71%) | 121 (71%) | 256 (72%) | 0.937 |
| ASA-PS classification |  |  |  | 0.352 |
| ASA-II | 109 (21%) | 31 (18%) | 78 (22%) | --- |
| ASA-III | 355 (67%) | 114 (67%) | 241 (67%) | --- |
| ASA-IV | 64 (12%) | 25 (15%) | 39 (11%) | --- |
| EUROSCORE II | 2 [3] | 3 [4] | 2 [2] | <0.001 |
| SOFA score D0 | 4 [3] | 6 [2] | 4 [2] | <0.001 |
| SAPS 3 score | 42 [12] | 46 [13] | 39 [11] | <0.001 |
| Comorbidities | 495 (94%) | 161 (95%) | 334 (93%) | 0.532 |
| Hypertension | 370 (70%) | 128 (80%) | 242 (72%) | 0.091 |
| Coronary artery disease | 363 (69%) | 129 (80%) | 234 (70%) | 0.018 |
| Diabetes mellitus | 196 (37%) | 69 (43%) | 127 (38%) | 0.303 |
| Heart failure | 171 (32%) | 58 (39%) | 113 (38%) | 0.945 |
| Preserved function | 82 (6%) | 26 (16%) | 56 (17%) | --- |
| Intermediate function | 49 (6%) | 18 (11%) | 31 (9%) | --- |
| Reduced function | 25 (6%) | 9 (6%) | 16 (5%) | --- |
| Very reduced function | 15 (6%) | 5 (3%) | 10 (3%) | --- |
| Unstable angina <90 days | 125 (24%) | 44 (27%) | 81 (24%) | 0.460 |
| Thrombocytopenia | 83 (16%) | 38 (22%) | 45 (13%) | 0.008 |
| Atrial fibrillation or flutter | 56 (11%) | 24 (15%) | 32 (10%) | 0.080 |
| Previous heart surgery | 53 (10%) | 16 (10%) | 37 (11%) | 0.701 |
| Stroke | 31 (6%) | 12 (7%) | 19 (5%) | 0.448 |
| CKD not requiring RRT | 30 (6%) | 9 (5%) | 21 (6%) | 0.918 |
| CKD requiring RRT | 10 (2%) | 4 (2%) | 6 (2%) | 0.866 |
| COPD | 29 (5%) | 9 (6%) | 20 (6%) | 0.860 |
| Anaemia (Hb <10 g dl^-1^) | 23 (4%) | 8 (5%) | 15 (5%) | 0.813 |
| Obstructive sleep apnoea | 23 (4%) | 7 (4%) | 16 (5%) | 0.827 |
| Rheumatic fever | 19 (4%) | 6 (4%) | 13 (4%) | 0.928 |
| Asthma | 14 (3%) | 3 (2%) | 11 (3%) | 0.564 |
| Active endocarditis | 12 (2%) | 9 (6%) | 3 (1%) | 0.003 |
| Active cancer | 9 (2%) | 5 (3%) | 4 (1%) | 0.159 |
| Laboratory tests before surgery | |  |  |  |
| Creatinine (mg/dL) | 1.16 [0.6] | 1.19 [0.7] | 1.14 [0.6] | 0.562 |
| Glucose (mg/dL) | 126 [63] | 141 [77] | 117 [50] | 0.004 |
| Haemoglobin (g/dL) | 13.3 [1.8] | 13.2 [2.0] | 13.4 [1.8] | 0.446 |
| Ionic calcium (mg/dL) | 1.18 [0.1] | 1.17 [0.1] | 1.18 [0.1] | 0.928 |

Note: Data are presented as mean [standard deviation] or as counts with percentages [n (%)]. *P*-values were calculated using the t-test for continuous variables and the chi-square test for categorical variables. Active endocarditis was defined as ongoing antibiotic therapy at the time of surgery. Heart failure was classified according to the left ventricular ejection fraction (LVEF): preserved function (LVEF ≥51%), intermediate function (LVEF 40–50%), reduced function (LVEF 30–39%), and very reduced function (LVEF ≤29%). Previous cardiac surgery was defined as any prior cardiac surgical procedure performed during a different hospitalisation.

Abbreviations: ASA-PS = American Society of Anesthesiologists Physical Status. BMI = body mass index; CKD = chronic kidney disease; COPD = chronic obstructive pulmonary disease; EUROSCORE = European System for Cardiac Operative Risk Evaluation. RRT = renal replacement therapy; SOFA = Sequential Organ Failure Assessment.

**Table AF 6.** Variables included in the univariate analyses of factors associated with mortality or severe postoperative complications.

| **Baseline** | **Comorbidities** | **Intraoperative** | **Postoperative** |
| --- | --- | --- | --- |
| **Demography** | Stroke** | Type of surgery performed** | **Development of complication** |
| Age* | Thrombocytopenia** | Percutaneous surgery | Vasoplegia** |
| BMI* | Cancer* | Open surgery | Anaemia* |
| Weight* | Diabetes mellitus* | Type of implanted valve** | Hyperglycaemia requiring intravenous insulin* |
| Male sex | Endocarditis* | Mechanical valve | Thrombocytopenia* |
| **Scores** | Hypertension* | Biological valve | Atrial fibrillation or flutter |
| ASA-PS classification* | Heart failure* | Anaesthesia administered* | **Laboratory tests D0** |
| EUROSCORE II* | Anaemia | General anaesthesia | pH** |
| **Laboratory tests before surgery** | Atrial fibrillation or flutter | Combined anaesthesia | Creatinine* |
| Creatinine* | Coronary heart disease | Sedation | Glucose* |
| Haemoglobin* | Previous heart surgery | Aortic cross clamp time* | Haemoglobin* |
| Ionic calcium | Unstable angina | Cardiopulmonary bypass time* | Ionic calcium |
| Glucose |  | Duration of surgery* | PaCO2 |
|  |  | Extubation at the end of surgery* | PaO2 |
|  |  | Classification of Intervention | **Scores** |
|  |  | Elective | SOFA score D0** |
|  |  | Urgent/emergency | SAPS 3 score* |
|  |  | Use of etomidate | Fluid balance |

Legend. *Covariates included in the final multivariable model. **Covariates independently associated with mortality or severe postoperative complications in the multivariable analysis.

Abbreviations: PaO₂ = arterial partial pressure of oxygen; PCO₂ = partial pressure of carbon dioxide; SOFA = Sequential Organ Failure Assessment.

**Table AF 7.** Descriptive statistics of variables requiring multiple imputation before inclusion in the univariate analyses of factors associated with mortality or severe postoperative complications.

| **Variable** | **Mean [SD] or n (%)** | **N^0^ of missing** | **Percentage of missing** |
| --- | --- | --- | --- |
| **Continuous variables** |  |  |  |
| Glucose D0 | 171 [54] | 261 | 49.4% |
| Creatinine D0 | 1.03 [0,48] | 83 | 15.7% |
| pH D0 | 7.35 [0.07] | 83 | 15.7% |
| Haemoglobin before surgery | 13.3 [1.8] | 71 | 13.4% |
| Duration of surgery | 221 [91] | 54 | 10.2% |
| **Binary variable** |  |  |  |
| Thrombocytopenia (Comorbidities) | 83 (16%) | 81 | 15.3% |

Note: When missingness in core variables exceeded 10%, multiple imputation by chained equations was applied following standard procedures. For variables with ≤10% missing data, complete-case analysis was performed.

Abbreviations: n (%) = counts with percentages; SD = standard deviation.

**Table AF 8.** Intraoperative data of included patients, stratified by the occurrence of mortality or severe postoperative complications.

|  | **All patients**  **(*n=*528)** | **Yes**  **(*n=*170)** | **No**  **(*n=*358)** | ***P*-value** |
| --- | --- | --- | --- | --- |
| **Anaesthesia administered** |  |  |  | <0.001 |
| General anaesthesia | 338 (64%) | 88 (52%) | 250 (70%) | --- |
| Combined anaesthesia | 176 (33%) | 79 (46%) | 97 (27%) | --- |
| Sedation | 14 (3%) | 3 (2%) | 11 (3%) | --- |
| **Complications during anaesthesia** | 33 (6%) | 19 (11%) | 14 (4%) | 0.001 |
| **Duration of surgery, min** | 221 [91] | 229 [92] | 217 [90] | 0.183 |
| **Total fluid intake (ml)** | 2652 [1638] | 2491 [1607] | 2731 [1649] | 0.043 |
| **Transfusion of blood products** | 118 (22%) | 45 (26%) | 73 (20%) | 0.117 |
| Red blood cells | 95 (18%) | 37 (22%) | 58 (16%) | --- |
| Platelets | 38 (7%) | 17 (10%) | 21 (6%) | --- |
| Fresh frozen plasma | 13 (2%) | 6 (4%) | 7 (2%) | --- |
| Cryoprecipitate | 12 (2%) | 4 (2%) | 8 (2%) | --- |
| **Type of surgery performed** |  |  |  | 0.275 |
| Elective | 430 (81%) | 143 (84%) | 287 (80%) | --- |
| Urgent/emergency | 98 (19%) | 27 (16%) | 71 (20%) | --- |
| **Urine output (ml)** | 1315 [1770] | 1323 [1833] | 1311 [1741] | 0.915 |

Note: Data are presented as mean [standard deviation] or as counts with percentages [n (%)]. *P*-values were calculated using the t-test for continuous variables and the chi-square test for categorical variables.

**Table AF 9.** Support interventions, medications, and devices used during the intraoperative period, stratified by type of surgery (open *vs* percutaneous).

|  | **All patients**  **(*n=*528)** | **Open-heart**  **(*n=*479)** | **Percutaneous**  **(*n=*49)** | ***P*-value** |
| --- | --- | --- | --- | --- |
| **Cardioplegic solutions** | 370 (70%) | 370 (77%) | 0 (0%) | --- |
| Not use any cardioplegia | 106 (20%) | 106 (22%) | 0 (0%) | --- |
| Solution modified / personalized by the surgical team | 103 (20%) | 103 (22%) | 0 (0%) | --- |
| St. Thomas | 100 (19%) | 100 (21%) | 0 (0%) | --- |
| Del Nido | 91 (17%) | 91 (19%) | 0 (0%) | --- |
| Histidine-Tryptophan-Ketoglutarate | 71 (13%) | 71 (15%) | 0 (0%) | --- |
| **Devices** |  |  |  |  |
| Cardiac output monitoring | 180 (34%) | 170 (35%) | 10 (20%) | 0.063 |
| Cell saver | 132 (25%) | 131 (27%) | 1 (2%) | <0.001 |
| Electroencephalogram-based monitoring | 240 (45%) | 224 (47%) | 16 (33%) | 0.182 |
| Thromboelastography or thromboelastometryc | 109 (21%) | 106 (22%) | 3 (6%) | 0.020 |
| **Extubation in the operating room** | 225 (43%) | 181 (38%) | 44 (90%) | <0.001 |
| **Medications** |  |  |  |  |
| Use of etomidate | 183 (34%) | 172 (35%) | 11 (22%) | 0.442 |
| Use of epsilon-aminocaproic acid | 67 (13%) | 65 (14%) | 2 (4%) | 0.205 |
| Use of tranexamic acid | 253 (48%) | 250 (52%) | 3 (6%) | <0.001 |

Note. Extubation in the operating room was defined as the absence of an endotracheal tube or supraglottic airway device (e.g., laryngeal mask) immediately before leaving the operating room. No tracheostomy was performed before surgery in any case. This definition also included patients who did not require airway devices during surgery and were therefore spontaneously breathing at the end of the procedure.

**Table AF 10.** Life-support interventions required on postoperative day 0, stratified by type of surgery (open *vs* percutaneous).

|  | **All patients**  **(*n=*528)** | **Open-heart**  **(*n=*479)** | **Percutaneous**  **(*n=*49)** | ***P*-value** |
| --- | --- | --- | --- | --- |
| **Vasoactive drugs** | 392 (74%) | 368 (77%) | 24 (49%) | 0.340 |
| Norepinephrine | 270 (51%) | 255 (53%) | 15 (31%) | 0.003 |
| Dobutamine | 201 (38%) | 189 (39%) | 12 (24%) | 0.040 |
| Nitroprusside | 54 (10%) | 48 (10%) | 6 (12%) | 0.625 |
| Nitroglycerin | 49 (9%) | 49 (10%) | 0 (0%) | 0.016 |
| Epinephrine | 33 (6%) | 33 (7%) | 0 (0%) | 0.061 |
| Vasopressin | 31 (6%) | 30 (6%) | 1 (2%) | 0.344 |
| Milrinone | 13 (2%) | 13 (3%) | 0 (0%) | 0.621 |
| Dopamine | 5 (1%) | 5 (1%) | 0 (0%) | --- |
| Levosimendan | 0 (0%) | 0 (0%) | 0 (0%) | --- |
| **Invasive mechanical ventilation** | 297 (56%) | 292 (61%) | 5 (10%) | <0.001 |
| **Circulatory assistance device** | 4 (1%) | 4 (1%) | 0 (0%) | --- |
| Intra-aortic balloon pump | 3 (1%) | 3 (1%) | 0 (0%) | --- |
| VA-ECMO | 1 (0%) | 1 (0%) | 0 (0%) | --- |
| **Renal replacement therapy** | 4 (1%) | 4 (1%) | 0 (0%) | --- |

Abbreviation: VA-ECMO = venoarterial extracorporeal membrane oxygenation.

**Table AF 11.** Life-support interventions required on postoperative days 0, 1, 2 and 3.

|  | **Day 0**  **(*n=*528)** | **Day 1**  **(*n=*522)** | **Day 2**  **(*n=*479)** | **Day 3**  **(*n=*332)** |
| --- | --- | --- | --- | --- |
| **Any life-support intervention** | 445 (84%) | 339 (65%) | 212 (44%) | 127 (38%) |
| **Vasoactive drugs** | 392 (74%) | 334 (63%) | 209 (44%) | 122 (37%) |
| Norepinephrine | 270 (51%) | 236 (45%) | 153 (32%) | 95 (29%) |
| Dobutamine | 201 (38%) | 181 (35%) | 110 (23%) | 58 (17%) |
| Nitroprusside | 54 (10%) | 24 (5%) | 10 (2%) | 2 (1%) |
| Nitroglycerin | 49 (9%) | 12 (2%) | 2 (0%) | 0 (0%) |
| Epinephrine | 33 (6%) | 14 (3%) | 7 (1%) | 4 (1%) |
| Vasopressin | 31 (6%) | 34 (7%) | 22 (5%) | 12 (4%) |
| Milrinone | 13 (2%) | 6 (1%) | 8 (2%) | 5 (2%) |
| Dopamine | 5 (1%) | 5 (1%) | 2 (0%) | 1 (0%) |
| Levosimendan | 0 (0%) | 0 (0%) | 0 (0%) | 0 (0%) |
| **Invasive mechanical ventilation** | 297 (56%) | 94 (18%) | 35 (7%) | 23 (7%) |
| **Circulatory assistance device** | 4 (1%) | 4 (1%) | 5 (1%) | 4 (1%) |
| Intra-aortic balloon pump | 3 (1%) | 3 (1%) | 4 (1%) | 3 (1%) |
| VA-ECMO | 1 (0%) | 1 (0%) | 1 (0%) | 1 (0%) |
| **Renal replacement therapy** | 4 (1%) | 7 (1%) | 6 (1%) | 4 (1%) |

Note. Daily decreases in n reflect ICU discharge or death; by the end of day 394 patients remained in the ICU. No patients were lost to follow-up. Because patients may receive multiple life-support interventions simultaneously, the sum of component counts does not equal the number receiving “any life-support intervention.”
