# Supplementary material for: Epidemiology, clinical outcomes and risk factors for mortality and severe postoperative complications in cardiac surgery patients admitted to intensive care units in Brazil: a multicentre observational study
Source: BJA Open. 2026 Aug 12;19:100580. doi: 10.1016/j.bjao.2026.100580 (PMC13499056; doi:10.1016/j.bjao.2026.100580)
Supplement: Supplementary file 2 [file mmc2.docx]

**BraSIS 2 Study Group**

Renato Carneiro de Freitas Chaves MD PhD, ^1*^ Nair Naiara Barros de Vasconcelos MD, ^1*^ Carolina de Moraes Pellegrino MD, ¹ Anna Clara Legal MS, ^1^ Guilherme Martins de Souza MD, ^1^ Veronica Neves Fialho Queiroz MD PhD, ^1^ Carmen Silvia Valente Barbas MD PhD, ^1^ Flávio Takaoka MD PhD, ^2^ Ricardo Luiz Cordioli MD PhD, ^1^ Sandrigo Mangini MD PhD, ^1^ Fabio de Vasconcelos Papa MD, ^3^ Hélio Penna Guimarães MD PhD, ^1^ Ary Serpa Neto MD MSc PhD, ^4^ Tiago Mendonça dos Santos MSc PhD, ^1^ Rogerio da Hora Passos MD PhD, ^1^ Frederico Toledo Campo Dall’Orto MD, ^5^ Eric Benedet Lineburger MD MSc PhD, ^6^ Vinícius Caldeira Quintão MD PhD, ^7^ Lucas Tramujas MD, ^8^ Caio Vinicius Gouvea Jaoude MD, ^9^ Karlos Alexandre de Sousa Vilarinho MD, ^10^ Brenno Cardoso Gomes MD MSc PhD, ^11^ Viviane Cordeiro Veiga MD PhD, ^12^ Erick César de Farias Albuquerque MD, ^13^ Ricardo Reinaldo Bergo MD, ^5^ Mauricio de Castro Gomes MD, ^5^ Gislayne Rogante Ribeiro MD, ^5^ Luis Guilherme Patricio MD, ^5^ Mariuska Perez Apolonio MD, ^5^ Júlia Turra Reichert MD, ^6^ Felipe Fernandes Ronsoni MD, ^6^ Ysys Bittencourt Martins Camargo MD, ^6^ Guilherme Hentges Fusiger MD, ^6^ Tales Ivan Freitas dos Santos MD, ^6^ Igor Almeida Cunha MD, ^6^ Kellen Nazário Pereira MD, ^6^ Andre Gulinelli MD, ^1^ Eduardo Paolinelli MD, ^1^ Fabio Barlem Hohmann MD PhD, ^1^ Felipe Galdino MD, ^1^ Felipe Vianna MD, ^1^ Luciano Ribeiro Pereira Silva MD, ^1^ Roberto Rabello Filho MD PhD, ^1^ Thais Dias Midega MD, ^1^ Vinicius Barbosa Galindo MD MSc, ^1^ Maria José Carvalho Carmona MD PhD, ^7^ Alexandra Patrícia Zilli Vieira Pharm MSc, ^7^ Maxim Goncharov MD, ^8^ Matheus Ligouri MD, ^8^ Marcelo Luz Pereira Romano MD, ^8^ Guilherme Nebó e Jambor MD, ^9^ Guilherme Benevenuto Hasebe MD, ^9^ Lara Beatriz Alves de Melo MD, ^9^ Orlando Petrucci Junior MD, ^10^ Matheus da Silva Passos MD, ^10^ Gustavo Lenci Marques MD MSc PhD, ^11^ Caroline Dourado Gomes MD, ^11^ Tom Pollard, ^14^ Adriano José Pereira MD PhD, ^1^ Thiago Domingos Corrêa MD PhD, ^1^* João Manoel Silva Júnior MD PhD ^1^*.

* RCFC, NNBV, and TDC JMSJr have contributed equally to this study.

^1^ Einstein Hospital Israelita, São Paulo, SP, Brazil.

^2^ Takaoka institute, São Paulo, SP, Brazil.

^3^ University of Toronto, Toronto, Canada.

^4^ University of Melbourne, Austin Hospital, Melbourne, Australia.

^5^ Hospital Maternidade e Pronto Socorro Santa Lúcia, Minas Gerais, MG, Brazil.

^6^ Hospital São José, Criciúma, SC, Brazil.

^7^ Academic Research Organization, Instituto do Coração, Hospital das Clínicas HCFMUSP, Faculdade de Medicina, Universidade de São Paulo, SP, Brazil

^8^ Instituto de Pesquisa do Hcor, São Paulo, SP, Brazil.

^9^ Instituto de Assistência Médica ao Servidor Público Estadual, São Paulo, SP, Brazil.

^10^ Universidade Estadual de Campinas, Campinas, SP, Brazil.

^11^ Hospital de Clínicas da Universidade Federal do Paraná, Paraná, PR, Brazil.

^12^ BP - A Beneficência Portuguesa de São Paulo, São Paulo, SP, Brazil.

^13^ Hospital Alberto Urquiza Wanderley, João Pessoa, PB, Brazil.

^14^ Massachusetts Institute of Technology, Cambridge, MA, United States.
